# Effect of dietary antioxidant-rich foods combined with aerobic training on energy metabolism in healthy young men

**DOI:** 10.3164/jcbn.18-40

**Published:** 2018-08-08

**Authors:** Maki Takami, Wataru Aoi, Hitomi Terajima, Yuko Tanimura, Sayori Wada, Akane Higashi

**Affiliations:** 1 Division of Applied Life Sciences, Graduate School of Life and Environmental Sciences, Kyoto Prefectural University, 1-5 Hangi-cho, Shimogamo, Sakyo-ku, Kyoto 606-8522, Japan; 2 Faculty of Human Health, Aichi-Toho University, 3-11 Heiwagaoka, Meito-ku, Nagoya 465-8515, Japan

**Keywords:** exercise training, antioxidant foods, aerobic metabolism, substrate oxidation, skeletal muscle

## Abstract

Although supplementation with several antioxidants has been suggested to improve aerobic metabolism during exercise, whether dietary foods containing such antioxidants can exert the metabolic modulation is unclear. This study aimed to investigate the effect of intake of the specific antioxidant-rich foods coupled with exercise training on energy metabolism. Twenty young healthy, untrained men were assigned to antioxidant and control groups: participants in the antioxidant group were encouraged to consume foods containing catechin, astaxanthin, quercetin, glutathione, and anthocyanin. All participants performed cycle training at 60% maximum oxygen consumption for 30 min, 3 days per week for 4 weeks. Maximum work load was significantly increased by training in both groups, while oxygen consumption during exercise was significantly increased in the antioxidant group only. There were positive correlations between maximum work load and fat/carbohydrate oxidations in the antioxidant group. Carbohydrate oxidation during rest was significantly higher in the post-training than that in the pre-training only in the antioxidant group. More decreased levels of serum insulin and HOMA-IR after training were observed in the antioxidant group than in the control group. This study suggests that specific antioxidant-rich foods could modulate training-induced aerobic metabolism of carbohydrate and fat during rest and exercise.

## Introduction

It has been shown that supplementation with several kinds of antioxidants contained in natural foods can improve energy metabolism during exercise in animals and humans.^(1–4)^ For example, supplementation with specific carotenoids, flavonoids, and glutathione activates aerobic metabolism associated with elevated mitochondrial function in skeletal muscle, a major metabolic organ.^(1,5–8)^ Because energy consumed in skeletal muscle is mainly supplied by carbohydrates and lipids, the activation of aerobic metabolism accelerates conversion from pyruvate to acetyl-CoA in glycolysis and utilization of fatty acids. This can suppress glycogen depletion and lactic acid generation during exercise. In addition, other antioxidants such as anthocyanin improve insulin sensitivity and glucose metabolism in skeletal muscle,^(9,10)^ which leads to increased supply of energy substrate from blood to the muscle. Therefore, the application of these supplements in the field of athletics and exercise therapy for health promotion is considered to have benefit, since the function of skeletal muscle is closely associated with athletic performance and with the development of metabolic disorders. It has been generally known that exercise training results in beneficial metabolic adaptations associated with insulin sensitivity and mitochondria activation,^(11)^ which could enhance athletic endurance performance and prevent/improve obesity and type 2 diabetes. Therefore, long-term supplementation with such compounds might provide some additive advantages in terms of the training-induced athletic and health adaptive benefits.

The effect of individual antioxidants in the form of powders, tablets, and capsules has been well examined. However, in the experimental conditions, these factors have been used in a quantity that is over 10-fold of the amount present in normal diets; such large amounts cannot usually be consumed from dietary sources. In contrast to be well known about the benefits of nutrients such as proteins, fats, and carbohydrates, whether phytochemicals such as antioxidants from whole foods are beneficial in the field of sports and nutritional science is unclear. The position stand of the related academic society recommend that athletes should satisfy their nutritional requirements through food rather than supplements.^(12)^ Indeed, phytochemicals from supplements (as powder, tablet, and capsule) and natural whole foods have different effects on the body, and continued consumption of supplements may also lead to negative effects.^(13,14)^ In contrast, whole foods contain not only the specific antioxidants but also various nutrients and other phytochemicals, which might have more merits compared to those observed by intake of a single factor. Several studies conducted in different backgrounds have suggested that combined intake of antioxidants often offers benefits that is difficult to be delivered by the intake of a single factor.^(15,16)^

On the basis of this information, we tested the hypothesis that dietary foods containing antioxidants that could boost aerobic metabolism may enhance the metabolic benefits induced by exercise training. This study focused on 5 food factors that have antioxidant effects, i.e., catechin, astaxanthin, quercetin, glutathione, and anthocyanin, which have been well documented. Here, we show that the intake of antioxidant-rich foods could modulate training-induced metabolic adaptation in young healthy men.

## Methods

### Participants

In total, 20 healthy young men who were not following a regular exercise regimen were recruited in this study. The characteristics of the participants are listed in Table 1. None of the participants had any signs, symptoms, or history of any overt chronic disease. None of them had a history of smoking and none were taking any medications or dietary supplements at the start of the study. The study was approved by the ethics committee of Kyoto Prefectural University, and all subjects signed a consent form after reading the design and protocol of the study (Permission No. 2012-46).

### Study design

**Fig. 1:**
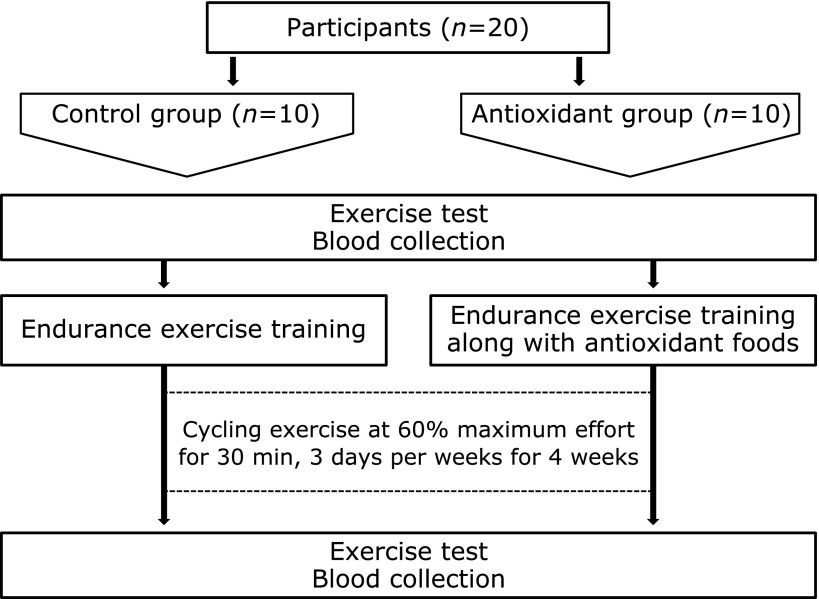
Study design. Twenty healthy young men who were not following a regular exercise regimen were divided into a control group (*n* = 10) and an antioxidant group (*n* = 10). All participants were subjected to supervised cycling training at 60% of maximum oxygen uptake for 30 min, 3 days per week for 4 weeks. Participants in the antioxidant group were provided nutritional education including information on typical antioxidants. Before and after this period, metabolic performance was measured and blood was collected for biochemical assays.

The participants were divided into a control group (*n* = 10) and an antioxidant group (*n* = 10), based on their physical characteristics (Fig. 1). No significant differences in the age, height, fat-free mass, body mass index, and endurance performance were observed between participants in the control and antioxidant groups before the intervention period. All participants were subjected to supervised cycling training at 60% of maximum oxygen consumption for 30 min, 3 days per week for 4 weeks. Participants in the antioxidant group were provided nutritional education including information on typical antioxidants that can improve metabolic capacity, i.e., catechin, astaxanthin, quercetin, glutathione, and anthocyanin. In addition, they were provided several antioxidant-rich foods, such as 60 g of salmon flakes and 90 g of canned salmon as sources of astaxanthin; 1,500 ml of green tea as a source of catechin; 3 onions and 3 apples as sources of quercetin; and 200 g of blueberry jam, 600 ml of purple vegetable juice, and 200 ml of grape juice as sources of anthocyanin every week, and consumed all of these foods. Food intake was recorded using the 3-day record method in the final week of the intervention period. Before and after this period, blood was collected after overnight fasting using a venous catheter, and serum samples were stored at −80°C for biochemical assays. On another day, respiratory gas during rest and exercise was analyzed to examine metabolic performance. Participants were asked to avoid caffeine 24 h before each measurement and to refrain from eating or drinking anything except water from 22:00 h till their measurement the next morning.

### Metabolic performance test

Before and after the intervention period, respiratory gas oxygen consumption and carbon dioxide production were measured to examine metabolic performance of each participant, using a breath-by-breath respirometer system (Aeromonitor AE310S; Minato, Osaka, Japan), during rest and incremental cycling exercise. To reduce breath-by-breath variation, these data were analyzed using a mean value obtained every 60 s. The respiratory exchange ratio and substrate utilization were calculated from the level of oxygen consumption and carbon dioxide production, as described previously.^(17)^

After the measurement at rest condition for 15 min, each participant exercised on the cycle ergometer (75XLII; Combi, Tokyo, Japan) at 60 rpm. The workload of the ergometer was gradually increased by 20 W every 2 min following 2 min of unloaded pedaling until oxygen consumption could not be increased further. The objective criteria for maximal effort included at least 2 of the following: (1) increased workload without corresponding increase in oxygen consumption; (2) respiratory exchange quotient equal to or greater than 1.10; and (3) a pedal cadence lower than 50 rpm despite maximal voluntary effort. As a result, the maximum oxygen consumption was determined and used for estimation of the intensity of 60% maximum effort.

### Dietary assessment

A dietary assessment was conducted to examine the intake of nutrients and antioxidants, at Week 4 of the intervention period. Participants were asked photographic recording of all foods that they consumed during the directed 3 days, along with dietary records of consecutive 3-day meals. Further, a hearing investigation for each participant was performed for complementary information of the meal and photographic records by a registered dietitian.

The amount of antioxidants ingested by the participants were calculated based on the presence of 3 mg of astaxanthin/100 g of salmon, 80 mg of catechin/100 g of green tea, 40 mg of quercetin/100 g of onion, 4 mg of quercetin/100 g of apple, and 5 mg of quercetin/100 g of blueberry components, according to a previous report.^(18,19)^ The amount of anthocyanin ingested by the participants was calculated based on the presence of 150 mg of anthocyanin/100 g of blueberries, 21 mg of anthocyanin/100 g of grape juice, and 45 mg of anthocyanin/100 g of purple vegetable juice, according to the information provided on the label of each product. The polyphenol content in the vegetable juice was calculated as the amount of anthocyanin. In addition, glutathione was calculated from the intake of spinatch (16 mg/100 g), cabbage (11 mg/100 g), cucumber (15 mg/100 g), tomato (29 mg/100 g), and pumpkin (12 mg/100 g), which are representative glutathione-containing foods, based on a previous report.^(20)^

### Measurement of blood parameters

Blood glucose concentrations were determined using the GluTest glucose monitor (Sanwa Kagaku Kenkyusho Co., Ltd., Nagoya, Japan). The serum samples were analyzed by FALCO Biosystems Corporation (Kyoto, Japan), and the triglyceride, non-esterified fatty acid (NEFA), and insulin levels were measured. Homeostasis model assessment-insulin resistance (HOMA-IR) levels were evaluated using the values of glucose and insulin. Irisin was detected using enzyme-linked immunosorbent assay, using a commercially available kit developed by Phoenix Pharmaceuticals Inc. (Burlingame, CA), according to the manufacturer’s instructions. As an index of oxidative state, the concentration of thiobarbituric acid-reactive substances (TBARS) was measured using the method described in the previous report.^(21)^ As an index of antioxidant capacity, the ferric reducing antioxidant power (FRAP) was measured using a commercially available kit (Cell Biolabs Inc., San Diego, CA), according to the manufacturer’s instructions.

### Statistical analysis

All data are reported as the mean ± SD. Because normal distribution was not obtained for all parameters, non-parametric analysis was used. Differences between the groups were evaluated using Mann-Whitney *U* and Wilcoxon signed-rank tests. Correlation between exercise performance and metabolic parameters was evaluated by Spearman’s correlation analysis. *P* values <0.05 were considered statistically significant.

## Results

### Physical characteristics and endurance parameters

 The body weight, body fat, and body mass index did not show significant changes between the groups by antioxidant intake or exercise training, although slightly decrease in body fat after training was observed in participants in the antioxidant group than in participants in the control group (Table 1). In the incremental exercise test, duration of exercise was significantly increased by 4 weeks of training in the control (*p* = 0.033) and the antioxidant groups (*p* = 0.003) (Table 1). Maximum workload was also significantly increased by training in both groups (*p* = 0.009, *p* = 0.009).

### Indirect calorimetric parameters

**Fig. 2:**
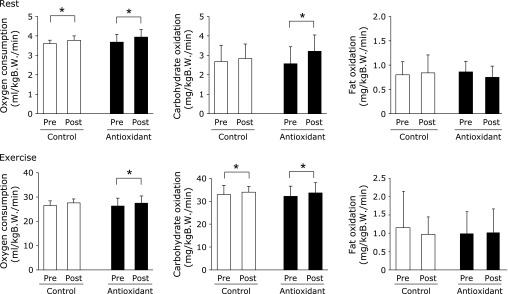
Metabolic performance in respiratory gas analysis before and after training in the control and antioxidant groups. Oxygen consumption, and carbohydrate and fat oxidations in the rest and exercise states. Values, obtained from 10 participants, are expressed as mean ± SD. *Significant difference at the level of *p*<0.05. B.W., body weight.

**Fig. 3:**
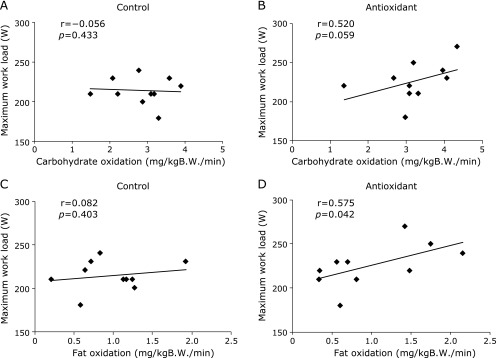
Relationship between substrates utilization and exercise performance. The correlation of carbohydrate oxidation during exercise with maximum workload after exercise training was plotted for the control (A) and antioxidant (B) groups. The correlation of fat oxidation during exercise with the maximum workload after exercise training was plotted for the control (C) and antioxidant (D) groups. Values are the mean ± SD obtained from 10 participants. B.W., body weight.

The mean value of oxygen consumption at rest was significantly increased by training in both groups (*p* = 0.037, *p* = 0.014), while there was no significant difference between the groups (Fig. 2). In contrast, the mean value of oxygen consumption during exercise was significantly increased after training compared with the mean value before training in the antioxidant group only (*p* = 0.014). Although carbohydrate utilization during exercise was significantly increased by training in both groups (*p* = 0.018, *p* = 0.006), it was significantly increased in the rest condition by training in the antioxidant group only (*p* = 0.023) (Fig. 2). No significant difference in fat utilization was noted either during rest or exercise conditions in both groups (Fig. 2). However, in the antioxidant group, there were positive correlations between fat oxidation during exercise and maximum workload after training (r = 0.575, *p* = 0.042), and between carbohydrate oxidation during exercise and maximum workload (r = 0.520, *p* = 0.059) (Fig. 3). No significant correlations with either carbohydrate (r = −0.056, *p* = 0.433) or fat (r = 0.082, *p* = 0.403) oxidations were observed in the control group.

### Blood biochemical parameters

In the fasting state, blood glucose level significantly increased by training in the antioxidant group (*p* = 0.030) but not in the control group (*p* = 0.452) (Table 2). Serum triglyceride level was significantly elevated by training in the control group only (*p* = 0.046). Serum insulin level showed no changes before and after training in any of the groups. However, the change (decrease) in insulin levels after training was significantly larger in the antioxidant group compared with the control group (*p* = 0.041). HOMA-IR levels also showed more changes in the antioxidant group than the control group (*p* = 0.099). The serum FRAP level was significantly elevated upon intervention only in the antioxidant group (*p* = 0.028). No significant differences in the levels of NEFA, irisin, and TBARS were observed before and after training in either of the groups (Table 2).

### Nutrient and antioxidant intake

The intake of astaxanthin (*p* = 0.002), catechin (*p* = 0.007), and quercetin (*p*<0.001) was found to be significantly larger in the antioxidant group than in the control group (Table 3). Anthocyanin intake was also greater in the antioxidant group than in the control group (*p* = 0.062). There were no significant differences in the intakes of vitamins A, C, and E between the groups. Intake of total energy, protein, carbohydrate was not significantly changed between both groups, fat intake was significantly decreased in the antioxidant group than in the control group (*p* = 0.002) (Table 3).

## Discussion

It is well known that exercise training exerts beneficial metabolic adaptations including improvement in insulin sensitivity and mitochondrial biogenesis in skeletal muscle, improvement of dyslipidemia, and reduction of body fat. Supplementation of several antioxidants such as astaxanthin, catechin, quercetin, glutathione, and anthocyanin can improve the energy metabolism, and could further prove beneficial in aerobic performance during acute and chronic exercise.^(5–8)^ However, it has not been clarified whether active intake of foods containing such antioxidants has additive effects on training-induced metabolic benefits. The present study revealed that intake of antioxidant-rich foods increased carbohydrate oxidation in rest state. In addition, this intervention showed higher oxygen uptake with modulating fat and carbohydrate oxidations during exercise although there were not additive effects on endurance performance. To the best of our knowledge, this is the first study to suggest that daily intake of foods containing specific antioxidants has some additive effects on aerobic metabolic adaptation induced by endurance training in humans.

Endurance exercise training induces increase in energy expenditure with improvement in aerobic metabolism of nutrients at rest.^(22,23)^ In this study, we confirmed that oxygen consumption during the rest state was increased in both groups. Along with this, carbohydrate oxidation was increased by training in the antioxidant group, while this effect was not observed in the control group. These results suggest that intake of antioxidant-rich foods could increase energy production via aerobic glucose metabolism. Because the participants consumed 200 g of steamed rice (74.2 g of carbohydrates) 2 h before respiratory gas analysis, their blood glucose level would be still higher, and their muscle and liver were filled with glycogen; therefore, carbohydrates tended to be used as the major energy substrate at this condition. Thus, the effect of antioxidant intervention might be mainly on the parameter related to the aerobic metabolism of carbohydrate but not fat. Serum insulin was also lower in the antioxidant group than in the control group, which supports the additive improvement of glucose metabolism induced by an antioxidant-rich diet.

In contrast to the rest condition, oxygen consumption during exercise was significantly increased in the antioxidant group only, which suggests that the intervention of antioxidant-rich foods promoted energy expenditure in not only the rest state but also the exercise state. During this time, although carbohydrate and fat oxidations did not differ between both groups, positive correlations were observed between the maximum workload and fat/carbohydrate oxidations in the antioxidant group; no such correlation was observed in the control group. These results suggest that aerobic metabolism of lipids and carbohydrates during exercise was higher in the subjects with a higher endurance after intervention of antioxidant-rich foods, which might explain the higher oxygen consumption during exercise. As another possibility, nonshivering thermogenesis might be elevated by the intake of some compounds contained in the foods. Previous studies have suggested that several muscle-secreted factors, referred to as myokines,^(24)^ could activate energy metabolism in adipose tissues and the liver.^(25–27)^ Interestingly, the expression/secretion of these myokines is upregulated by a key metabolic modulator, peroxisome proliferator-activated receptor-coactivator-1α, the production of which is induced by catechin, astaxanthin, and quercetin. Although a muscle-secreted factor, irisin was not found to be changed by either training or food intake in the present study, other factors may explain the elevation of oxygen uptake.

In this study, participants actively consumed antioxidant-rich foods such as astaxanthin, catechin, quercetin, anthocyanin, and glutathione, along with the training program. Astaxanthin is a xanthophyll carotenoid present in algae, fish, and crustaceans. We and other researchers have previously shown that astaxanthin stimulates lipid metabolism in skeletal muscle through mitochondrial activation.^(1,28)^

The flavonoids catechin and quercetin widely exist in fruit and vegetables including onions, apples, and various kinds of teas, and can activate aerobic metabolism and accelerate mitochondrial biogenesis.^(2,7,8,28)^ In addition, it has recently been shown that glutathione, a factor contained in natural foods including fruit and vegetables, accelerates aerobic metabolism via mitochondrial biogenesis in skeletal muscle.^(6)^ This factor is synthesized and widely exists in the human body, although dietary intake can lead to effects different from that produced by the endogenous factor. Meanwhile, anthocyanins present in purple fruits including berries exert beneficial effects on insulin dependent- and independent-glucose metabolism in the muscle. Taken together, these antioxidants can facilitate aerobic metabolism of glucose and lipids by activating the biogenesis of mitochondrial and glucose uptake in the muscle, which might lead to higher aerobic utilization of nutrients and lower levels of fasting insulin and HOMA-IR in the post-training period than in the pre-training period in the antioxidant group.

During the intervention period, the intake of astaxanthin, catechin, quercetin, and anthocyanin in the antioxidant group was markedly higher than that in the control group. However, the amount of antioxidants ingested is typically lower in comparison with the amount present in these factors in the form of supplements used in previous studies. In this study, the intake of astaxanthin was 2.8 mg, which was slightly lower than the amount of 4.0–6.0 mg consumed in previous studies.^(29–31)^ Quercetin intake was 97.5 mg, which corresponded to approximately one-tenth of the 1,000 mg amount in quercetin supplements used in the previous study,^(32)^ while catechin intake was 797.3 mg, which was similar to the intake of 405–625 mg reported in previous studies.^(33,34)^ Anthocyanin intake was 157.5 mg, which was lower than the intake of 320 mg in the form of anthocyanin supplements reported in the previous study.^(35)^ Taken together, the amount of the majority of antioxidants was lower than the amount contained in the supplements used in previous studies. Such large amount cannot be obtained from foods, e.g., 1,000 mg of quercetin corresponds to 2.5 kg and 12.5 pieces of onion. Thus, the amount of antioxidants that the participants in this study consumed was already close to the upper limit of the range when the antioxidants were consumed from the foods. Indeed, the serum antioxidant capacity was increased by the intake of antioxidant-rich foods, suggesting that the amount of those foods was enough for those ingredients to exert antioxidant effects. Exercise training is known to induce endogenous antioxidant enzymes associated with sirtuin-3 in blood monocytes.^(36)^ Thus, our result suggests that the intervention of antioxidant-rich foods accelerated the training-induced improvement of antioxidant capacity. In addition, this study cannot be compared with the previous studies that used a single ingredient in the form of a supplement, because whole foods contain not only antioxidants but also other phytochemicals; thus, a combination of all ingredients, even in small amounts, may exert an additive effect on the individual.

Although there is some debate regarding the intake of dietary antioxidants during exercise, a high dose of dietary antioxidants combined with an exercise regimen can counteract the oxidative stress that induces beneficial effects brought about by moderate exercise. For example, administration of vitamin C (1,000 mg/day) and vitamin E (400 IU/day) along with exercise training significantly ameliorated improvements in glucose infusion rates during a hyperinsulinemic (euglycemic) clamp, and adaptation of endurance capacity.^(37,38)^ The negative effects of antioxidant vitamins would result from their capacity to reduce the exercise-induced expression of the key transcription factors involved in nutrient metabolism. However, some antioxidants used in this study accelerate energy metabolism and insulin sensitivity induced by exercise, by increasing the level and activity of key modulators.^(1,6,7,39)^ Therefore, this may account for the specific actions of each compound in addition to the antioxidant properties. The effectiveness of the ingredients may also differ according to the gender and individual characteristics of the participants, and the mode of ingestion. Although the serum antioxidant capacity was increased by the intake of antioxidant-rich foods, we cannot conclude whether the beneficial effects were mediated by the antioxidant function.

This study has some additional limitations. First, the intensity of exercise training was set at 60% of maximum effort, which corresponds to moderate intensity; thus, endurance parameters could not be markedly increased by the training. Therefore, in order to validate the effect of antioxidant-rich foods on endurance performance, further studies with higher work load, duration, and frequency of exercise training that enhances markedly maximum oxygen uptake and work load are necessary. Secondly, it is unknown whether the beneficial results observed after the intake of antioxidant-rich foods were attributed to a single antioxidant ingredient or the combined action of all ingredients contained in foods. Therefore, further large-scale research is warranted to establish the optimum method of intake, the quantity and quality of the foods to be ingested, and the timing of their intake, in order to understand the purpose of using each food or food component and the physiological changes brought by exercise.

In conclusion, the present study showed that active intake of foods containing certain antioxidants induced carbohydrate oxidation after training, during conditions of rest. Although endurance performance after training did not differ between participants in the antioxidant and control groups, a significant positive correlation between maximum workload and oxidation of lipids during exercise was observed in the antioxidant group only. In addition, a decrease in serum insulin level after training was observed in participants in the antioxidant group only. These results suggest that intake of dietary antioxidant-rich foods combined with exercise training could modulate nutrient aerobic metabolism during rest and exercise. Further research is required to examine whether these findings can be generalized to the larger public.

## Author Contributions

MT, WA, and AH conceived and designed research. MT, HT, YT, and SW conducted experiments. MT, WA, and HT analyzed data. MT and WA wrote the manuscript with input from other authors. All authors reviewed and approved the manuscript.

## Figures and Tables

**Table 1:** Participant characteristics before and after training

	Control				Antioxidant			*p* values^#^
	Pre	Post	*p* values*		Pre	Post	*p* values*	
Age (years)	20.8 ± 0.3				21.4 ± 0.4			
Height (cm)	170.6 ± 1.8				170.6 ± 3.0			
Body weight (kg)	60.2 ± 1.2	60.3 ± 1.3	0.476		63.0 ± 3.0	63.1 ± 3.0	0.323	0.367
Body fat (%)	12.8 ± 0.8	13.1 ± 1.0	0.342		13.3 ± 1.5	12.7 ± 1.4	0.166	0.128
Bodymass index	20.7 ± 0.3	20.7 ± 0.3	0.417		21.6 ± 0.7	21.6 ± 0.7	0.380	0.325
Maximum work load (watt)	206 ± 6	214 ± 5	0.009		218 ± 8	226 ± 8	0.009	0.500
Duration of exercise (min)	18.0 ± 0.6	19.6 ± 0.6	0.033		19.5 ± 0.8	21.0 ± 0.8	0.003	0.312

Values are the mean ± SD obtained from 10 participants. *p values in the comparing between before and after exercise training between the groups. ^#^*p* values in the comparing of the change in the levels before and after exercise training between the groups.

**Table 2:** Blood biochemical parameters before and after training

	Control				Antioxidant			*p* values^#^
	Pre	Post	*p* values*		Pre	Post	*p* values*	
Glucose (mg/dl)	85.3 ± 2.4	85.4 ± 2.7	0.452		86.4 ± 1.8	91.2 ± 2.2	0.030	0.154
Triglyceride (mg/dl)	54.2 ± 7.7	82.5 ± 18.9	0.046		74.0 ± 11.9	82.8 ± 10.9	0.142	0.470
NEFA (mEq/L)	0.46 ± 0.07	0.31 ± 0.06	0.063		0.62 ± 0.13	0.50 ± 0.06	0.288	0.470
Insulin (µU/ml)	4.63 ± 0.53	5.78 ± 1.19	0.297		4.99 ± 0.53	4.18 ± 0.40	0.107	0.041
HOMA-IR	0.99 ± 0.13	1.26 ± 0.30	0.101		1.08 ± 0.13	0.95 ± 0.11	0.143	0.099
Irisin (ng/ml)	13.5 ± 3.1	19.0 ± 6.3	0.297		19.8 ± 5.0	14.9 ± 3.3	0.107	0.117
TBARS (pmol/mg)	1.09 ± 0.13	1.16 ± 0.12	0.179		1.20 ± 0.16	1.09 ± 0.14	0.193	0.128
FRAP (µM)	94.0 ± 13.0	98.2 ± 17.7	0.314		94.0 ± 11.3	102.7 ± 12.2	0.028	0.486

Values are the mean ± SD obtained from 10 participants. *p values in the comparing between before and after exercise training between the groups. ^#^*p* values in the comparing of the change in the levels before and after exercise training between the groups.

**Table 3:** Amount of nutrients and antioxidants consumed by participants in the control and antioxidant groups

	Control	Antioxidant	*p* values
Total energy intake (kcal)	2,149 ± 98	2,005 ± 141	0.325
Protein (g)	81.4 ± 3.9	70.9 ± 6.2	0.066
Fat (g)	72.2 ± 6.3	48.8 ± 4.9	0.002
Carbohydrate (g)	280 ± 13	309 ± 24	0.182
Astaxanthin (mg)	0.03 ± 0.03	2.76 ± 0.85	0.002
Catechin (mg)	174 ± 112	797 ± 182	0.007
Quercetin (mg)	12.9 ± 3.2	97.5 ± 21.3	<0.001
Anthocyanin (mg)	4.5 ± 4.5	157.5 ± 46.6	0.062
Glutathione (mg)	19.4 ± 4.8	26.0 ± 6.1	0.273
Vitamin A (µg)	1,687 ± 224	2,244 ± 997	0.182
Vitamin C (mg)	236 ± 41	286 ± 53	0.248
Vitamin E (mg)	31.0 ± 9.8	46.4 ± 25.0	0.470

Values are the mean ± SD obtained from 10 participants.

## Abbreviations

FRAP: ferric reducing antioxidant power
HOMA-IR: homeostasis model assessment-insulin resistance
NEFA: non-esterified fatty acid
TBARS: thiobarbituric acid-reactive substances
